# Dataset for hierarchical tetramodal-porous architecture of zinc oxide nanoparticles microfluidically synthesized via dual-step nanofabrication

**DOI:** 10.1016/j.dib.2022.108137

**Published:** 2022-04-11

**Authors:** Su-Eon Jin, Sung-Joo Hwang, Hyo-Eon Jin

**Affiliations:** a College of Pharmacy, Yonsei University, Incheon 21983, South Korea; b College of Pharmacy, Ajou University, Suwon 16499, South Korea

**Keywords:** Zinc oxide nanoparticles, Dual-step nanofabrication, Three-dimensional architecture, Hierarchical porosity, Bioinks

## Abstract

Zinc oxide (ZnO) nanoparticles (NPs) have been applied as high-performance intelligent materials to create a hierarchical multimodal-porous architectures for application in biomedical research fields [1]. They were microfluidically synthesized via dual-step nanofabrication compared to the conventional particles including ZnO NPs synthesized at single-pot macroscale, nanosized ZnO, and hybrid ZnO. The physicochemical properties were characterized, including morphology, particle size distribution, atomic composition, crystallinity, purity, reactant viscosity, surface charge, photocatalysis, photoluminescence, and porosity. A hierarchical multimodal-porous three-dimensional (3D) architecture of ZnO NPs was generated and optimized on the solid plate substrate of cellulose paper sheet after solvent evaporation. The dataset provides the nanomaterial design and architecture generation of ZnO NPs, explaining multi-physics phenomena in association with performance optimization processes.

## Specifications Table

**Table:** 

Subject	Materials Science: Nanotechnology
Specific subject area	Nanoparticle synthesis, Surface modulation, and Characterization
Type of data	Table Image Graph Figure
How data were acquired	Field emission-scanning electron microscope (FE-SEM) with energy-dispersive X-ray spectrometer (EDS) (S-4300SE, Hitachi, Co. Ltd., Japan) Fourier-transform infrared (FT-IR) spectrometer (Bruker Vertex 80v, Bruker Optics, Inc., Billerica, MA, USA) Viscometer (DV2T, AMETEK Brookfield, Middleboro, MA, USA) Field emission-transmission electron microscope (FE-TEM, JEM-2100F, Jeol, Co. Ltd., Peabody, MA, USA) Zeta potential and particle size analyzer (ELS-Z, Otsuka Electronics Co., Ltd., Tokyo, Japan) X-ray photoelectron spectrometer (XPS, K-Alpha, Thermo Scientific, Thermo Fisher Scientific Inc., Hillsboro, OR, USA) Raman spectrometer (Horiba LabRam HR Evolution, Horiba, Ltd., UK) Ultraviolet (UV)-visible (vis) spectrophotometer (DU730, Beckman Coulter, Inc., Brea, CA, USA) UV-vis spectrometer (Jaz System, Ocean Optics, Inc., Orlando, FL, USA) with a softwawre (Spectra Suite, Ocean Optics, Inc.) Synergy H1 hybrid multi-mode fluorescence reader (BioTek Instrumetns, Inc., Winooski, VT, USA) with Gen 5 software (BioTek Instruments, Inc.) Adsorption analyzer (Tristar and ASAP 2020 Plus Physisorption, Micromeritics Instrument Corp., Norcross, GA, USA) Image J (NIH, National Institutes of Health, Bethesda, Maryland, USA) Minitab® (ver.17.2.1, Eretech, Inc., Anyang, Gyeonggi-do, Korea)
Data format	Raw and analyzed
Parameters for data collection	Zinc oxide (ZnO) nanoparticles (NPs) were microfluidically synthesized and further solidified comparing with single-pot ZnO synthesis at macroscale. Nanosized and multiscale ZnO particles were also used as conventional products.
Description of data collection	Physicochemical characterization was performed in terms of morphology, particle size distribution, atomic composition, crystallinity, purity, reactant viscosity, surface charge, photocatalysis, photoluminescence, and porosity. Hierarchical multimodal-porous architecture and texture of zinc oxide (ZnO) nanoparticles (NPs) were analyzed after dropping serially on solid plate substrate of cellulose filter paper.
Data source location	Primary data sources: Ajou University Institution: College of Pharmacy, Ajou University City/Town/Region: Suwon Country: Korea Secondary data sources: Yonsei University Institution: College of Pharmacy, Yonsei University City/Town/Region: Incheon Country: Korea
Data accessibility	Repository name: Mendeley Data Data identification number: 10.17632/sh4d5z2szx.1 Direct URL to data: https://data.mendeley.com/datasets/sh4d5z2szx/1
Related research article	S.E. Jin, S.J. Hwang, H.E. Jin, Hierarchical tetramodal-porous architecture of zinc oxide nanoparticles microfluidically synthesized via dual-step nanofabrication, Mater. Des. 215 (2022) 110486. https://doi.org/10.1016/j.matdes.2022.110486.

## Value of the Data

• Microfluidically synthesized zinc oxide (ZnO) nanoparticles (NPs) generate hierarchical multimodal-porous three-dimensional (3D) architecture for advanced biomedical applications.
• Physicochemical characterization provides the design information of ZnO NPs and their architectures, based on the theoretical and experimental analyses of the dataset.
• Researchers, such as physicists, chemists, biologists, (bio)engineers, and clinicians, can have of particular interest to use the dataset for comparison with other ZnO materials and the further explanation of fabrication and architecture generation.
• The dataset can be beneficial for the development of fundamental NP evaluation protocols regarding the characterization of physicochemical properties and generation of 3D architecture of NPs with hierarchical multimodal porosity.

## 1 Data Description

Zinc oxide (ZnO) nanoparticles (NPs) have been explored as multifunctional nanomaterials finding applications in advanced biomedical research fields 2,3. Their hierarchical porosity with multi-modality of micropores (<2 nm), mesopores (2–50 nm), and macropores (>50 nm) can be designed to enhance their performance in sensing, energy harvesting, and photocatalysis when used in medical devices and therapeutics, due to advanced mass transfer and exchange 4,5. Currently, ZnO NPs can be synthesized using physical, chemical, and biological methods to meet the required characteristics regarding morphology, particle size distribution, crystallinity, purity, surface charge, photocatalysis, photoluminescence, and porosity [6]. A microfluidic device (MFD) is an advanced equipment in a miniaturized lab system for the sophisticated and controllable synthesis of ZnO NPs [7,8]. MFD integration into conventional methods can be beneficial for engineered NP generation accompanied with the designed and targeted physicochemical characteristics. Microfluidically synthesized ZnO NPs present hierarchical porosity with tetra-modality generating three-dimensional (3D) architecture, compared to the conventional particles of ZnO NPs synthesized using single-pot at macroscale, nanosized ZnO (nano-ZnO), and multiscale ZnO (hybrid-ZnO) [1,8].

**Fig. 1:**
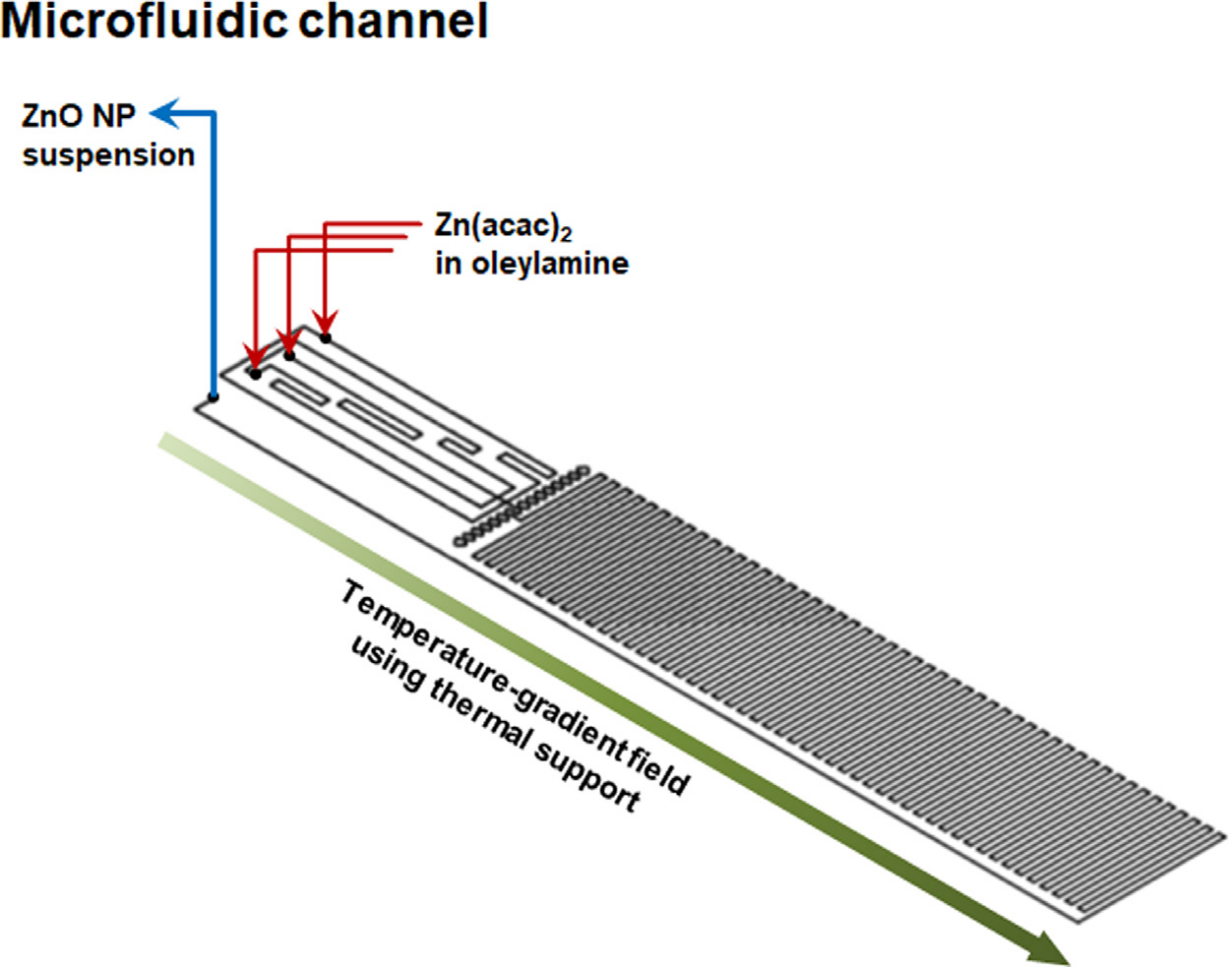
Microfluidic arrangement of microflow with heat transfer for ZnO NP synthesis in the microfluidic chemical reactor chip.

Fig. 1 shows a microfluidic chemical reactor chip used for the microfluidic synthesis of ZnO NPs providing microflow with heat transfer within microfluidic channels. In the microfluidic chemical reactor chip, there were three inlets and one outlet with a long serpentine channel connected to a syringe pump controlling at 0.1 mL⋅min^−1^. Its total volume was sized at 250 μL.

**Fig. 2:**
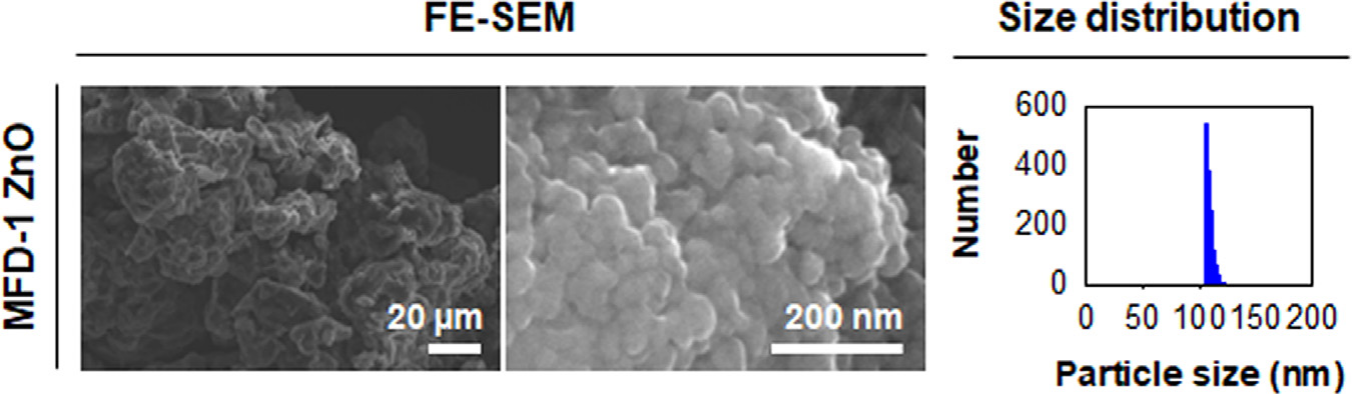
Microfluidically synthesized ZnO NPs: FE-SEM images, and the size distribution plot of MFD-1 ZnO.

Fig. 2 gives morphology and particle size distribution of MFD-1 ZnO using FE-SEM with EDS, which presented one of formulae for developing microfluidically synthesized ZnO NPs. MFD-1 ZnO was synthesized with Zn(acac)_2_ in oleylamine at 1.67% (3 mL) for optimizing the microfluidic synthesis formulae of ZnO NPs.

**Fig. 3:**
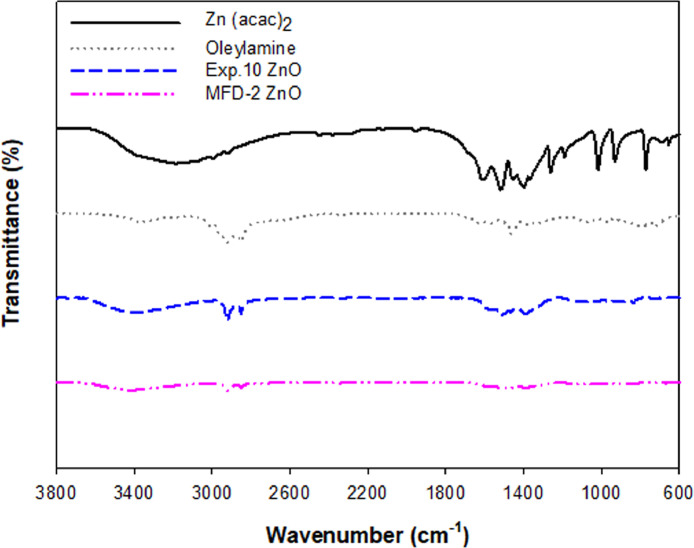
FT-IR spectra of Zn(acac)_2_, oleylamine and MFD-2 ZnO. Exp.10 ZnO (one of Box-Behnken design formulae at 10 of experimental run order in macroscale synthesis of ZnO NPs, See Table 1) was used as a control nanomaterial compared to MFD-2 ZnO.

Fig. 3 displays the purity of microfluidically synthesized ZnO NPs, specifically MFD-2 ZnO, using FT-IR spectroscopy. MFD-2 ZnO was developed at 2.5% of Zn(acac)_2_ in oleylamine. After the ethanol rinsing of ZnO NPs in a dual-step synthesis process, the purity after the maximum removal of residual amines was confirmed in the FT-IR spectra. Zn(acac)_2_, oleylamine, and Exp.10 ZnO of single-pot ZnO synthesis at macroscale in a Box-Behnken design (See Table 1) were compared with MFD-2 ZnO.

**Table 1:** Box-Behnken design formulae in ZnO NP synthesis using single-pot at macroscale.

		Independent variables		
Standard order	Experiment (Run order)	Zn(acac)_2_ (g)	Oleylamine (mL)	Ethanol (mL)
1	Exp. 1	0.050	1.0	150
2	Exp. 2	0.400	1.0	150
13	Exp. 3	0.225	3.5	150
8	Exp. 4	0.400	3.5	250
15	Exp. 5	0.225	3.5	150
9	Exp. 6	0.225	1.0	50
10	Exp. 7	0.225	6.0	50
3	Exp. 8	0.050	6.0	150
7	Exp. 9	0.050	3.5	250
14	Exp. 10	0.225	3.5	150
5	Exp. 11	0.050	3.5	50
12	Exp. 12	0.225	6.0	250
6	Exp. 13	0.400	3.5	50
11	Exp. 14	0.225	1.0	250
4	Exp. 15	0.400	6.0	150

**Fig. 4:**
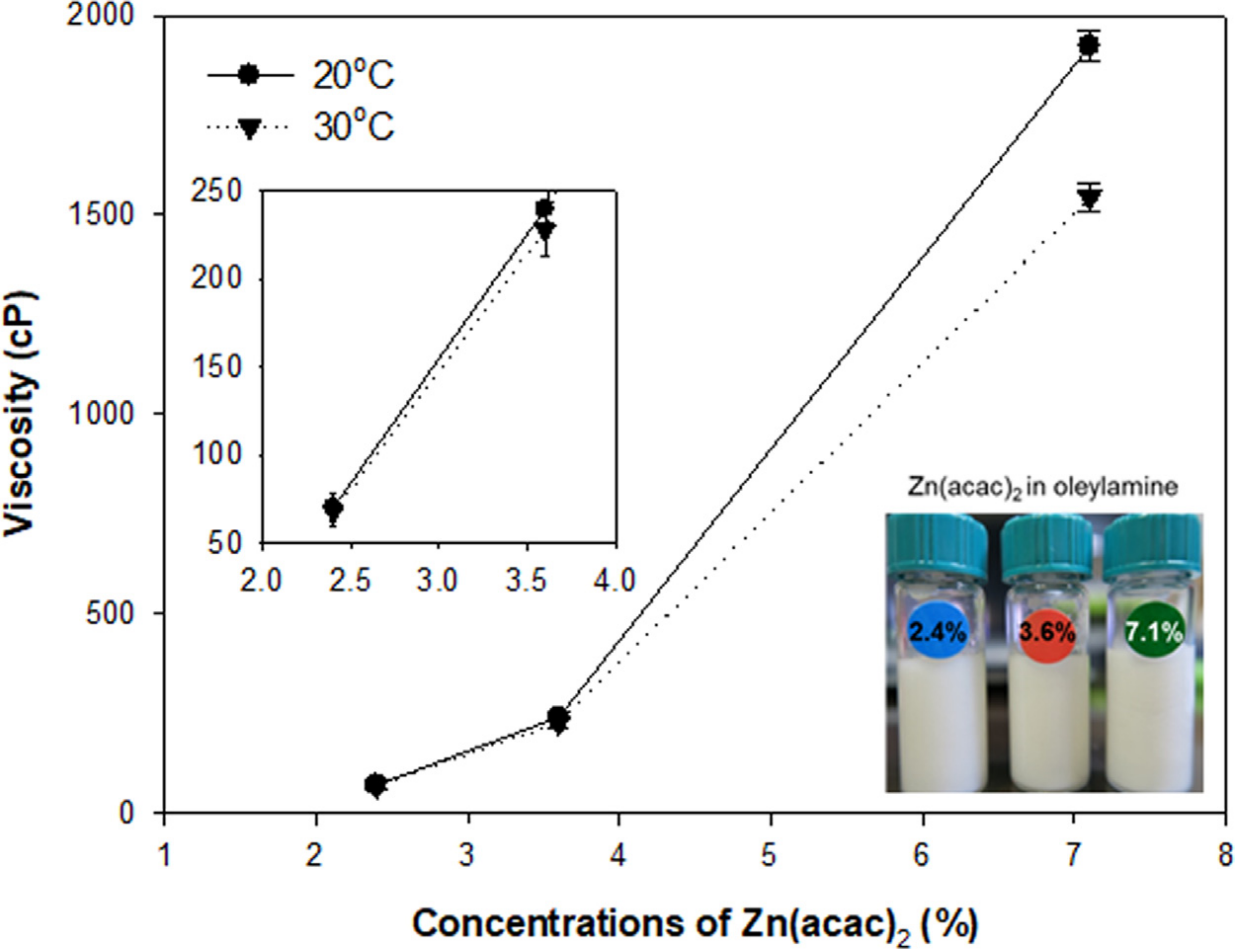
Viscosity of Zn(acac)_2_ in oleylamine. Zn(acac)_2_ was used at 2.4%, 3.6%, and 7.1% (w⋅v^−1^) in oleylamine.

Fig. 4 offers the viscosity of Zn(acac)_2_ in oleylamine at 2.4%, 3.6%, and 7.1% (w⋅v^−1^) at 20 °C and 30 °C as a controllable factor for the microfluidic synthesis of ZnO NPs, predicting the microflow in the internal microfluidic channel of the chip. The image presents the Zn(acac)_2_ solutions in oleylamine.

**Fig. 5:**
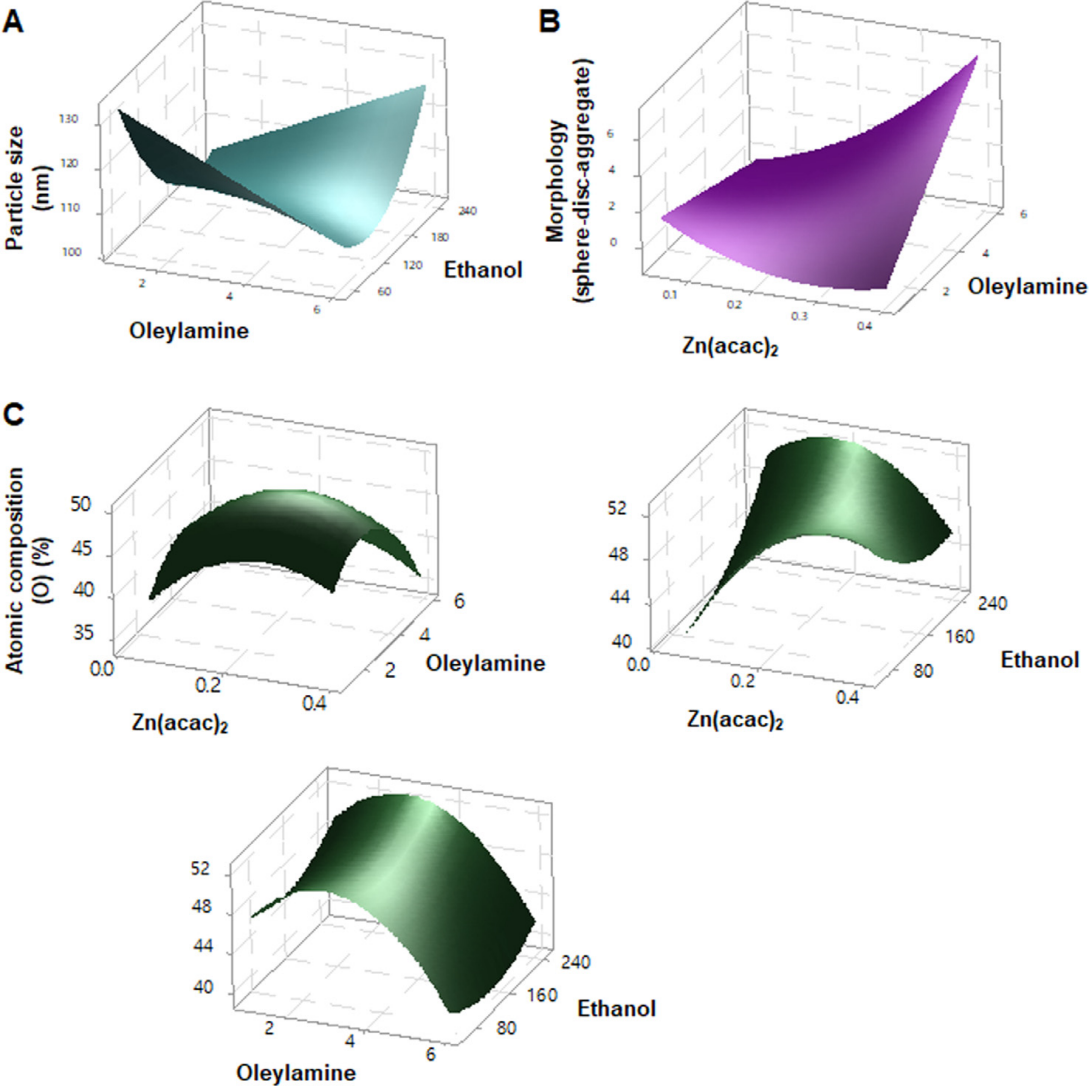
3D surface plots: effects of reactant amounts on (A) particle size, (B) morphology, and (C) atomic composition of ZnO NPs in macroscale synthesis.

Fig. 5 provides 3D surface plots illustrating reactant interactions affecting the particle size, morphology, and atomic composition of ZnO NPs during single-pot synthesis at macroscale based on the Box-Behnken design formulae (See Table 1).

**Fig. 6:**
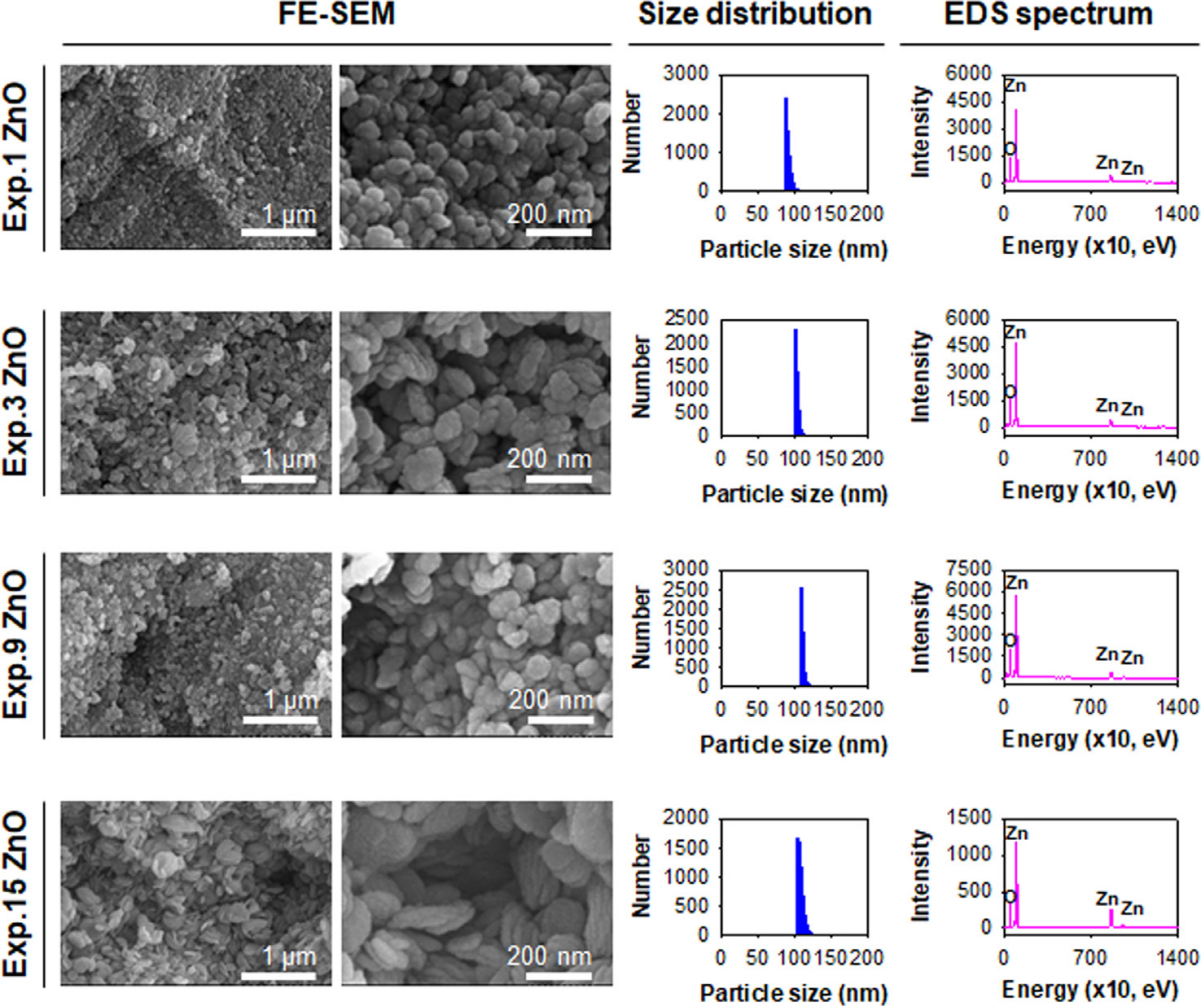
Representative results of synthesized ZnO NPs in Box-Behnken design: FE-SEM images, particle size distribution plots, and EDS profiles. In the EDS profiles, Zn was detected at 1,030 eV, 8,640 eV, and 9,570 eV and O was detected at 530 eV. Each experiment (Exp.) numbered ZnO was one of Box-Behnken design formulae (See Table 1) at experimental run orders 1, 3, 9, and 15 in the macroscale synthesis of ZnO NPs.

Fig. 6 presents the FE-SEM images, particle size distribution plots, and EDS spectra of representative ZnO NPs after single-pot synthesis at macroscale suggesting their morphology, particle size distribution, and atomic composition. The formulae at 1, 3, 9, and 15 of run orders among experimental order numbers in Box-Behnken design (See Table 1) are displayed (Exp.1, Exp.3, Exp.9, and Exp.15 ZnOs).

**Fig. 7:**
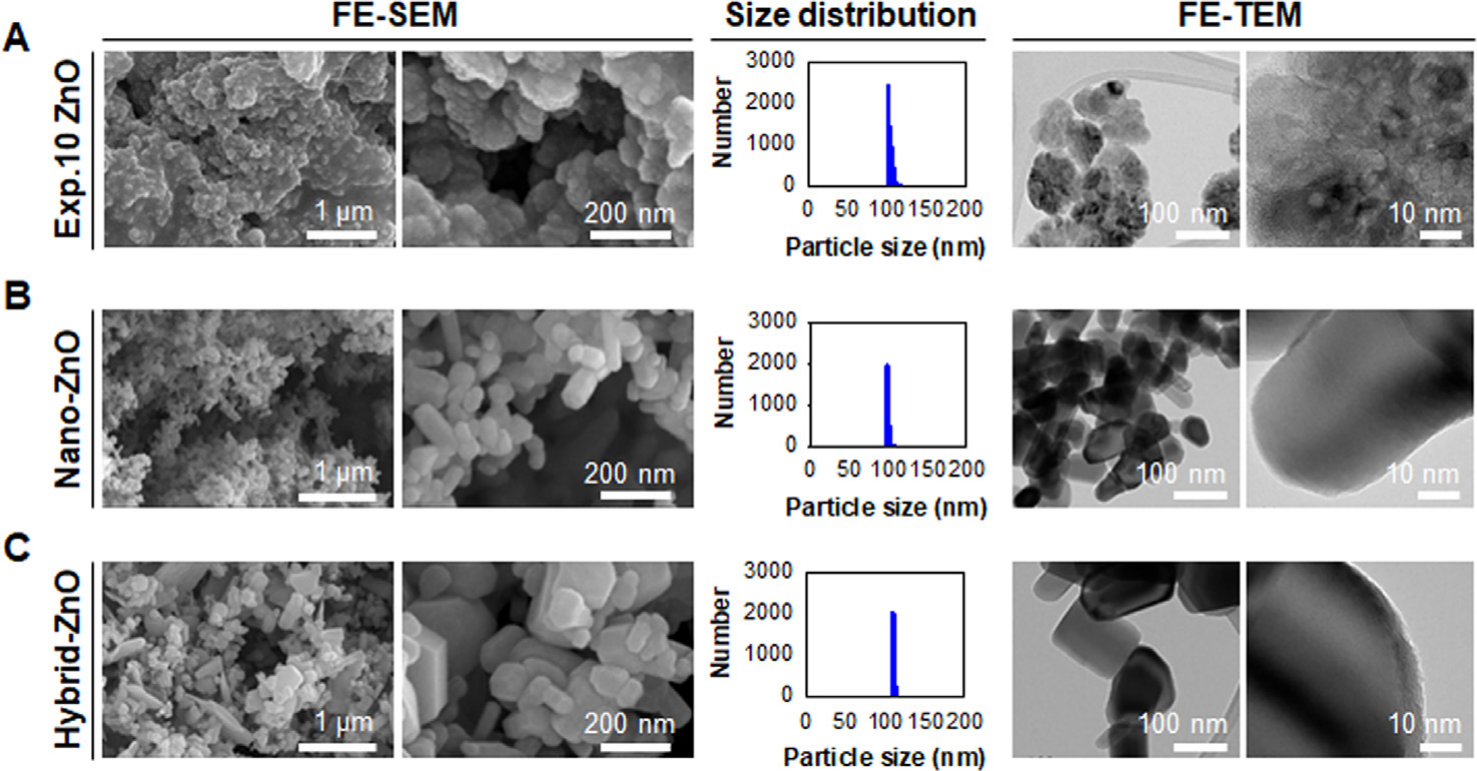
FE-SEM images, particle size distribution plots, and FE-TEM images of conventional ZnO particles: (A) Exp.10 ZnO, (B) nano-ZnO, and (C) hybrid-ZnO.

Fig. 7 shows the FE-SEM images, particle size distribution plots, and FE-TEM images of conventional ZnO particles including ZnO NPs at 10 of the experimental run order synthesized using single-pot at macroscale (Exp.10 ZnO, See Table 1), nano-ZnO, and hybrid-ZnO. Exp.10 ZnO was synthesized with 0.225 g of Zn(acac)_2_ and 3.5 mL of oleylamine, and rinsed with 150 mL of ethanol. They provide the morphology, particle size distribution, and crystal structure of conventional ZnO particles compared with those of microfluidically synthesized ZnO NPs.

**Fig. 8:**
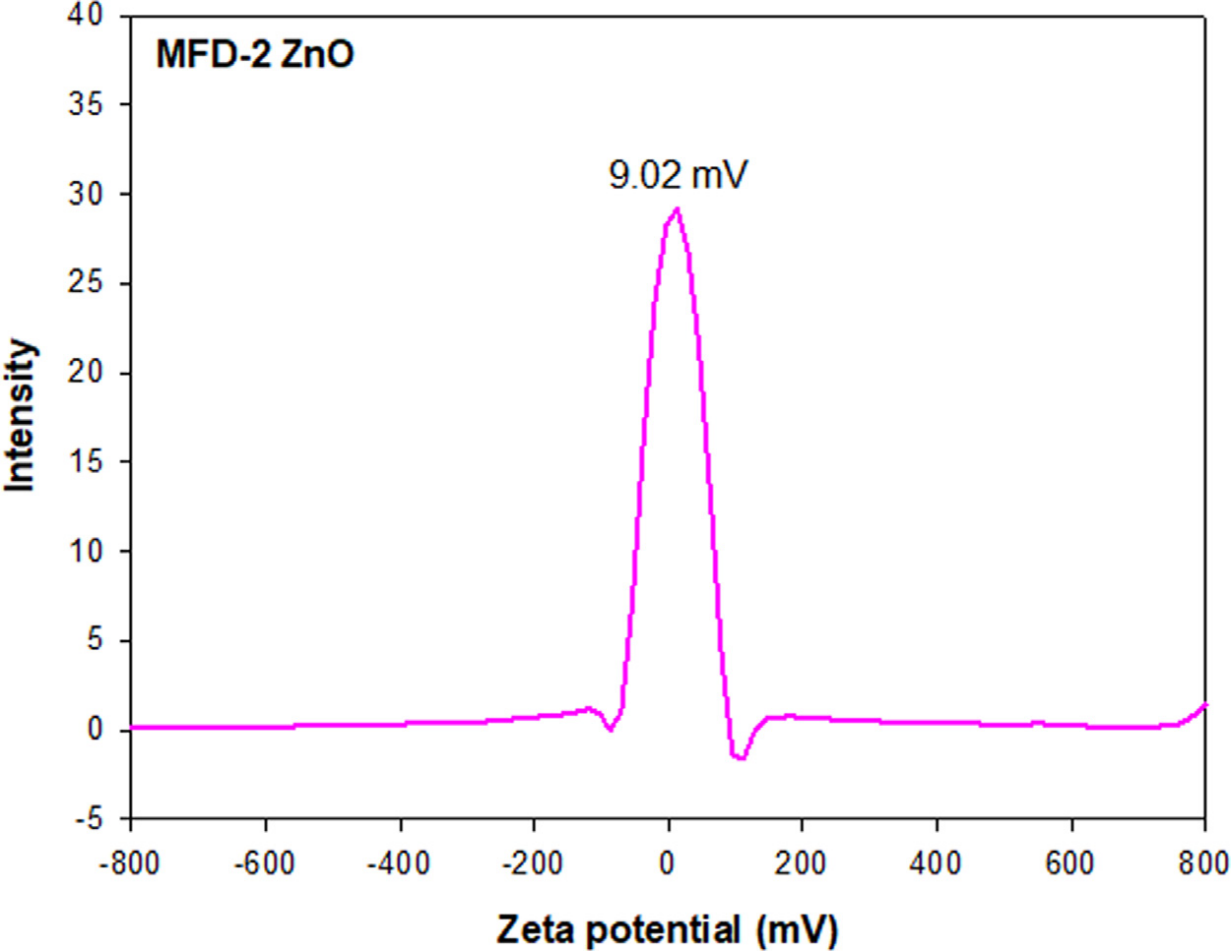
Zeta potential distribution of MFD-2 ZnO. Average zeta potential value was 9.02 mV.

Fig. 8 presents the zeta potential distribution plot of MFD-2 ZnO with an average value of 9.02 mV. MFD-2 ZnO was suspended in ethanol and appropriately diluted with fresh ethanol to reach the operating intensity range for zeta potential measurement.

**Fig. 9:**
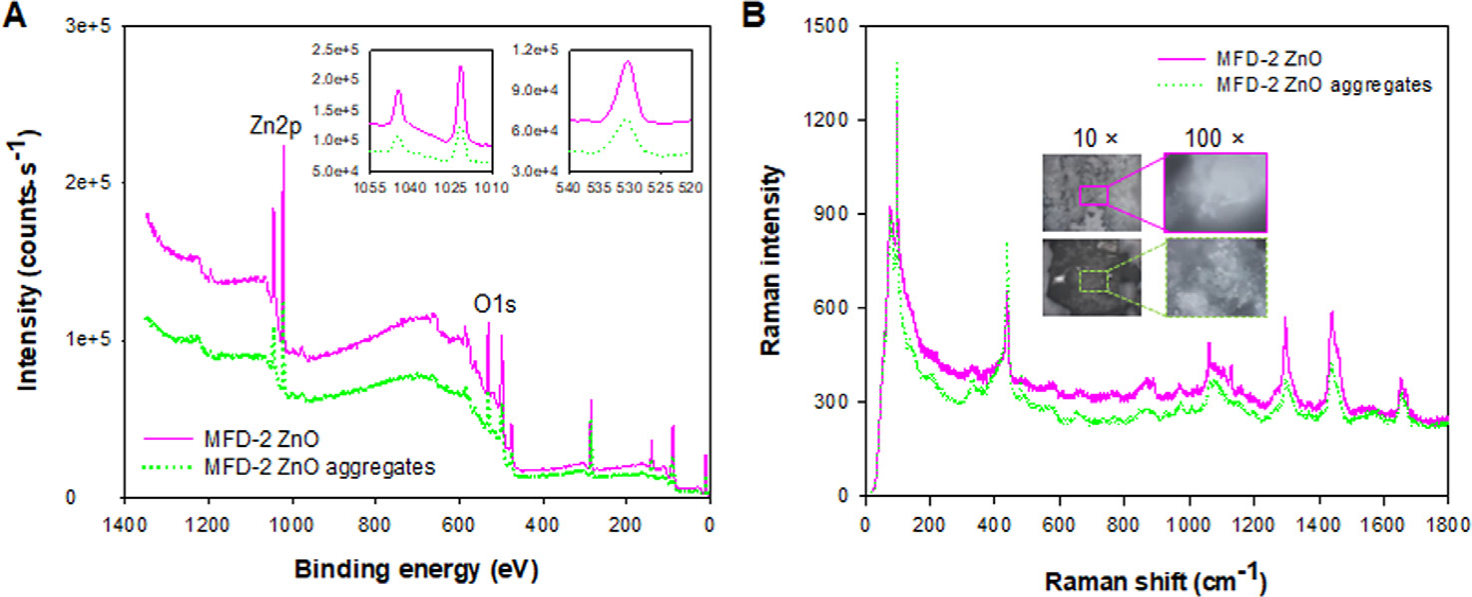
(A) XPS and (B) Raman profiles of MFD-2 ZnO and the aggregates.

Fig. 9 shows the XPS and Raman profiles of the MFD-2 ZnO and MFD-2 ZnO aggregates. MFD-2 ZnO aggregate powders were prepared after dripping the hexane suspensions of MFD-2 ZnO on a glass slide and drying at room temperature for 24 h.

**Fig. 10:**
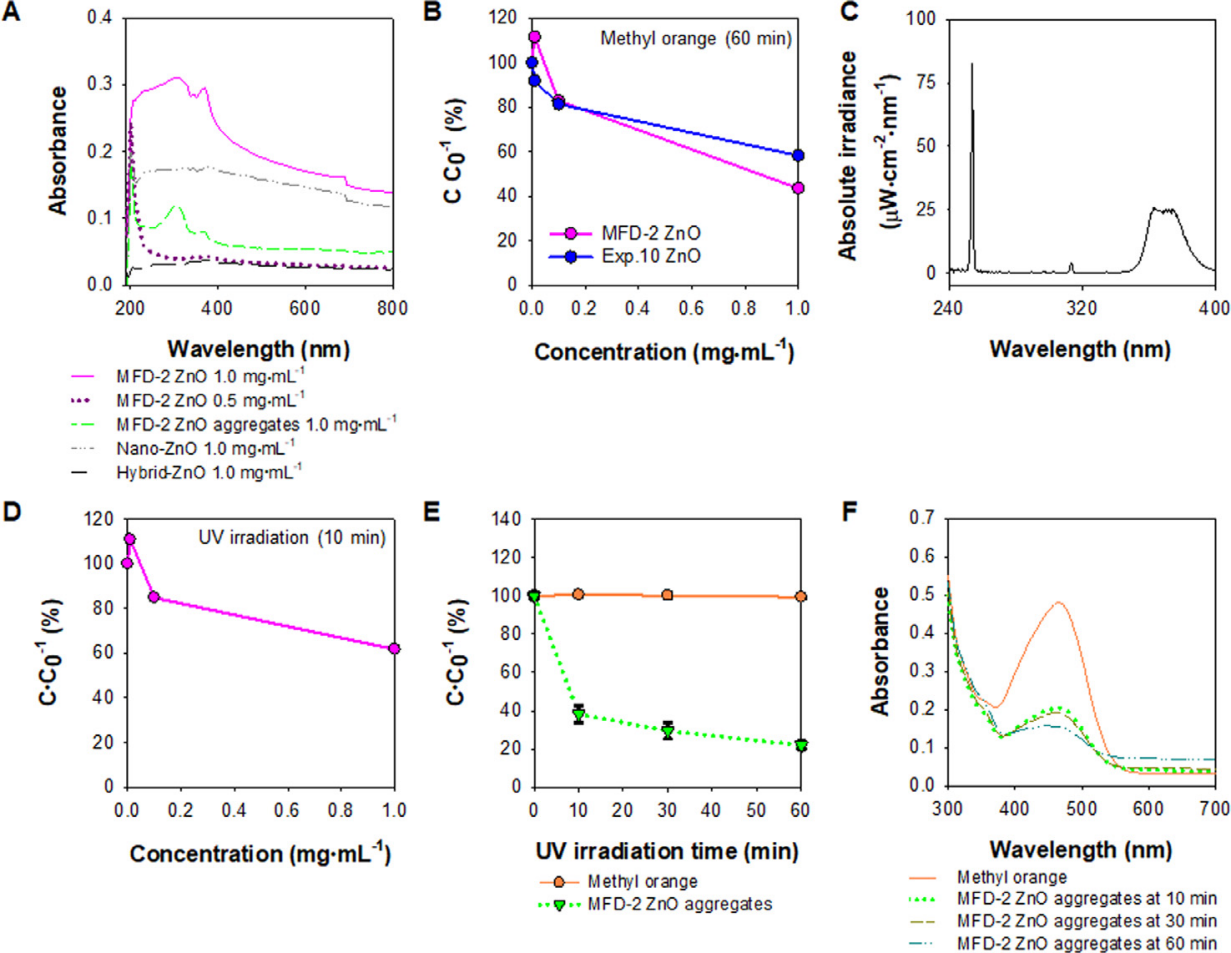
Photocatalytic characteristics of MFD-2 ZnO and the aggregates: (A) UV-vis absorption; (B) sorption of methyl orange for 60 min; (C) dual UV spectrum of UV-A and UV-C in CBD; photocatalytic degradation profiles of methyl orange (0.01 mg⋅mL^−1^) using (D) MFD-2 ZnO (0.01 – 1.0 mg⋅mL^−1^) and (E) MFD-2 ZnO aggregates (1.0 mg⋅mL^−1^) after dual UV irradiation (MFD-2 ZnO, 10 min; MFD-2 ZnO aggregates, 10, 30, and 60 min); and (F) absorbance levels of methyl orange after photocatalytic degradation using MFD-2 ZnO aggregates after dual UV irradiation for 10, 30, and 60 min. Methyl orange was detected at 464 nm.

Fig. 10 displays the photocatalytic properties of MFD-2 ZnO and the aggregates based on the ultraviolet (UV) absorption and sorption of methyl orange (0.1 mg⋅mL^−1^) for chemical removal. A dual UV spectrum containing UV-A (315–400 nm) and UV-C (100–280 nm) was obtained using a collimated beam device (CBD). The photocatalytic degradation of methyl orange by MFD-2 ZnO and the aggregates were monitored under dual UV irradiation.

**Fig. 11:**
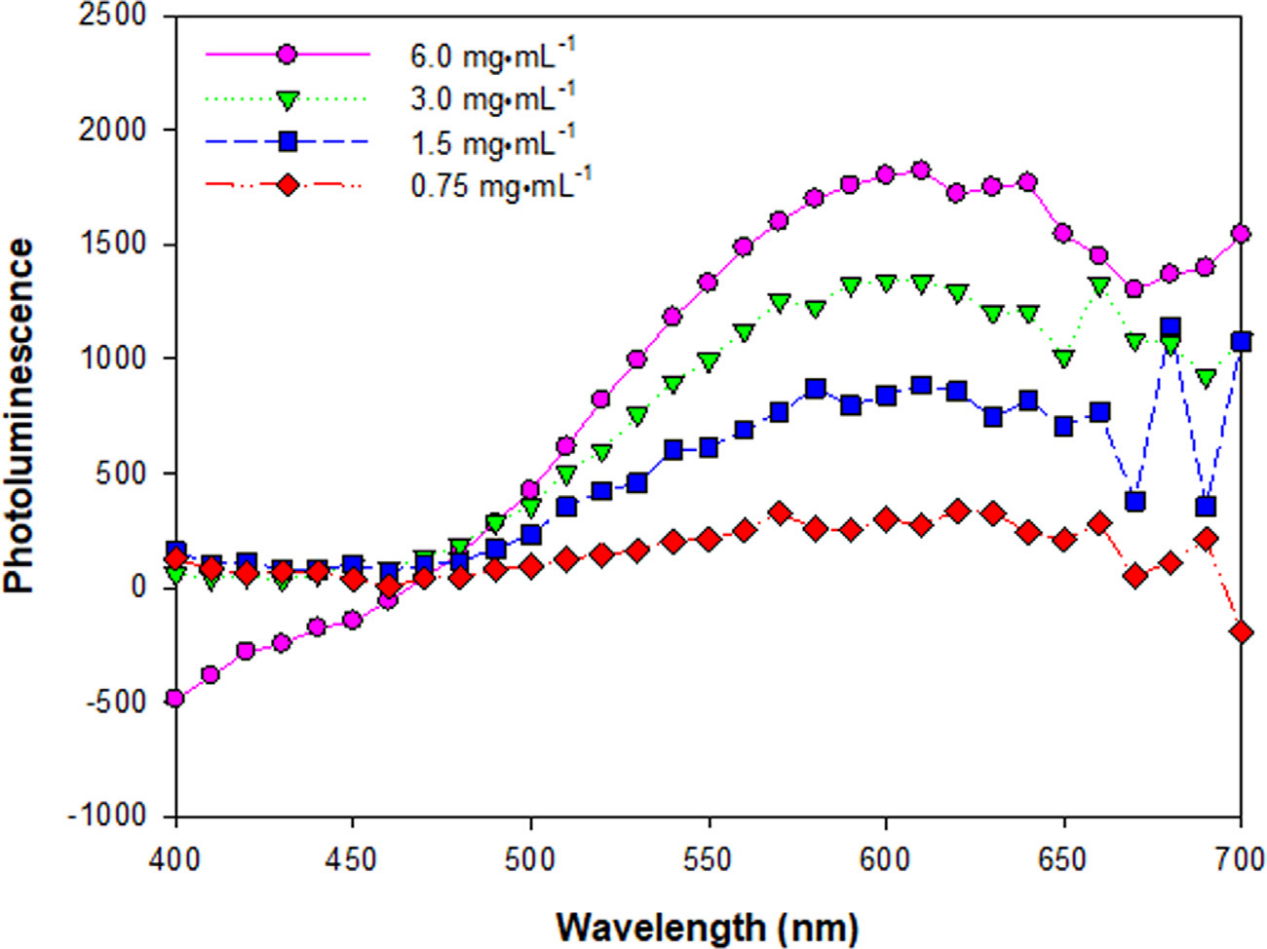
PL of MFD-2 ZnO at 0.75–6.0 mg⋅mL^−1^.

Fig. 11 provides the photoluminescence (PL) of MFD-2 ZnO at the range of 0.75–6.0 mg⋅mL^−1^. The excitation wavelength was 358 nm, and the emission wavelength was at the range of 400–700 nm.

**Fig. 12:**
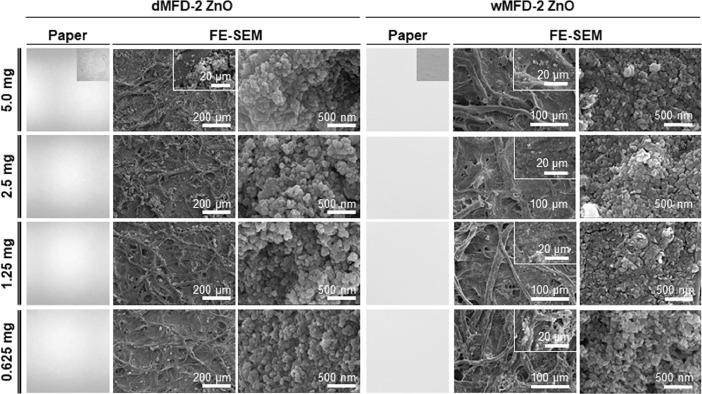
Immobilization of MFD-2 ZnO on cellulose paper sheet at 0.625–5.0 mg. Two types of ZnO NPs were used: dMFD-2 (dried) and wMFD-2 (ethanol-wetted) ZnOs. The cellulose paper sheet and FE-SEM images were obtained after immobilization. MFD-2 ZnO covered the solid plate substrate surface or generated the hierarchical porous architecture depending on NP ethanol wetting and amounts. Specifically, the wMFD-2 ZnO started forming the hierarchical porous architecture at 0.625 mg.

Fig. 12 shows the immobilization of MFD-2 ZnO on a cellulose paper sheet depending on the NP ethanol wetting and NP amounts (0.625–5.0 mg). In association with NP ethanol wetting, two types of MFD-2 ZnO were used for immobilization on the cellulose paper sheet: dMFD-2 ZnO as the dried form and wMFD-2 ZnO as the ethanol-wetted form. Cellulose paper sheet photos and the FE-SEM images of immobilized MFD-2 ZnO on the cellulose paper sheet were obtained, providing information on the NP covering or hierarchical porous architecture generation of NP network.

Table 1 lists the single-pot synthesis of ZnO NPs at macroscale in Box-Behnken design to investigate each interaction of reactants and rinsing solvent for synthesis of ZnO NPs. The independent variables of Zn(acac)_2_, oleylamine, and ethanol were selected as 0.05–0.4 g, 1.0–6.0 mL, and 50–250 mL, respectively, for 15 experiments.

Table 2 presents the surface characteristics, such as pore size (nm or Å), pore volume (cm^3^⋅g^−1^), and surface area (m^2^⋅g^−1^) of MFD-2 ZnO compared to those of Exp.10 ZnO (See Table 1), nano-ZnO, and hybrid-ZnO after mesopore and micropore analyses based on Brunauer-Emmett-Teller (BET) theory.

Table 3 lists the atomic composition of MFD-2 ZnO compared to those of conventional ZnO particles including Exp.10 ZnO (See Table 1), nano-ZnO, and hybrid-ZnO monitored in the EDS profiles.

**Table 2:** ZnO particle porosity.

Particles	Pore size (nm or Å)	Pore volume (cm^3^⋅g^−1^)	Surface area (m^2^⋅g^−1^)
*Mesopore analysis* (nm)			
MFD-2 ZnO†	19.48	0.127	26.1 ± 0.14
Exp.10 ZnO‡	33.43	0.163	19.5 ± 0.06
Nano-ZnO#	14.33	0.0487	13.6 ± 0.02
Hybrid-ZnO§	18.26	0.0339	7.4 ± 0.01
*Micropore analysis* (Å)			
MFD-2 ZnO	17.92	0.1504	83.9072

† MFD-2 ZnO: microfluidically synthesized ZnO NPs.

‡ Exp.10 ZnO: macroscale-synthesized ZnO NPs, one of representative Box-Behnken design formulae at 10 of the experimental run order (See Table 1).

# Nano-ZnO: nanosized ZnO NPs.

§ Hybrid-ZnO: multiscale ZnO particles.

**Table 3:** Atomic compositions of ZnO particles.

	Atomic compositions (%)	
ZnO particles	Zn	O
MFD-2 ZnO†	57.2 ± 6.6	42.8 ± 6.6
Exp.10 ZnO‡	57.9 ± 6.5	42.1 ± 6.5
Nano-ZnO#	59.0 ± 5.9	41.0 ± 5.9
Hybrid-ZnO§	55.3 ± 2.4	44.7 ± 2.4

† MFD-2 ZnO: microfluidically synthesized ZnO NPs.

‡ Exp.10 ZnO: macroscale-synthesized ZnO NPs, one of representative Box-Behnken design formulae at 10 of experimental run order (See Table 1).

# Nano-ZnO: nanosized ZnO NPs.

§ Hybrid-ZnO: multiscale ZnO particles.

## 2 Experimental Design, Materials and Methods

### 2.1 Microfluidic synthesis of ZnO NPs in a chemical reactor chip via dual-step nanofabrication

Intrinsic nanoporous ZnO NPs were nucleated and solidified via dual-step reactions of zinc acetylacetonate hydrate [Zn(acac)_2_] (Sigma) and oleylamine (3 mL; 1.67% for MFD-1 ZnO, 2.5% for MFD-2 ZnO, and 5.0% for MFD-3 ZnO, w⋅v^−1^) in a microfluidic process (microfluidic chemical reactor chip, Dolomite, Blacktrace Holdings Ltd., Royston, UK) at 80 °C for 30 min, along with a further heating process at 150 °C for 1 h. Preheating, mixing, and synthesis processes were continuously performed in the MFD. The flow rate was 0.1 mL⋅min^−1^ controlled using a syringe pump. After cooling to room temperature, the mixture was poured into ethanol (100 mL) and the resulting white precipitates were rinsed with ethanol (Merck), and dried. The premade aggregates of microfluidically synthesized ZnO NPs were generated after dripping the NP suspensions in hexane (Sigma) on glass slide and drying at room temperature for 24 h.

### 2.2 Single-pot macroscale synthesis of ZnO NPs in Box-Behnken design

The interactions of Zn(acac)_2_, oleylamine, and ethanol were monitored using a Box-Behnken design (Minitab® ver.17.2.1, Eretech, Inc., Anyang, Korea). Zn(acac)_2_ was weighed (0.05–0.4 g) and dispersed in oleylamine (1.0–6.0 mL). A mixture of Zn(acac)_2_ and oleylamine was heated at 80 °C for 30 min and then kept at 150 °C for 1 h. After cooling to room temperature, it was poured into ethanol (50–250 mL). The white precipitates were rinsed with ethanol three times and were dried. Experiment (Exp.) numbered ZnO represents a Box-Behnken design formulae followed by run order.

### 2.3 Field emission-scanning electron microscopy (FE-SEM) with energy-dispersive X-ray spectroscopy (EDS)

Morphology was monitored using field emission-scanning electron microscopy (FE-SEM, S-4300SE, Hitachi, Co. Ltd., Japan) operated at an acceleration voltage of 15.0 kV. Particle size distribution was displayed from obtained FE-SEM images using ImageJ (NIH). EDS analysis was also performed on the particle surface to quantitatively determine the element composition.

### 2.4 Fourier-transform infrared (FT-IR) spectroscopy

The removal of residual amines from ZnO NPs was determined by FT-IR spectroscopy. The powdered samples were mixed with potassium bromide (KBr) and subjected to a pressure to produce a disc. IR spectra region at 3,800–600 cm^−1^ were recorded on a FT-IR vacuum spectrometer (Bruker Vertex 80v, Bruker Optics, Inc., Billerica, MA, USA). For each spectrum, the frequencies for all sharp bands were accurate to 0.01 cm^−1^.

### 2.5 Viscosity measurement

Reactant viscosity levels in ZnO NP synthesis were measured using a viscometer (DV2T, AMETEK Brookfield, Middleboro, MA, USA). Data were analyzed with Rheocalc T software (version 1.2.19, AMETEK Brookfield). Various concentrations of Zn(acac)_2_ in oleylamine were ranged at 2.4–7.1% (w⋅v^−1^). Temperature was maintained at 20 °C and 30 °C during the experiments.

### 2.6 Field emission-transmission electron microscopy (FE-TEM)

Field emission-transmission electron microscopy (FE-TEM, JEM-2100F, Jeol, Co. Ltd., Peabody, MA, USA) was performed at acceleration voltages of 200.0 kV.

### 2.7 Zeta potential measurement

Zeta potential values of ZnO NPs were measured at room temperature using a light scattering (ELS-Z, Otsuka Electronics Co., Ltd., Tokyo, Japan) with standard cell. ZnO NP dispersions in ethanol were diluted with fresh ethanol prior to analysis to reach the appropriate intensity for the measurements. Huckel equation was used to calculate zeta potential values for conversion, and the average zeta potential (mV) was determined in ELS-Z software (Otsuka Electronics Co., Ltd.).

### 2.8 X-ray photoelectron spectroscopy (XPS)

The atomic compositions of ZnO NPs and the aggregates were determined using XPS (K-Alpha, Thermo Scientific, Thermo Fisher Scientific Inc., Hillsboro, OR, USA) with a monochromated Al K-alpha source.

### 2.9 Raman spectroscopy

ZnO NPs and aggregates were placed on a glass slide and were subjected to Raman spectrometry (Horiba LabRam HR Evolution, Horiba, Ltd., UK) with a laser (wavelength, 532 nm). Raman spectra were recorded with five acquisitions and three accumulations.

### 2.10 Ultraviolet (UV)-visible (Vis) scanning

Optical properties of ZnO NPs were scanned using UV-vis spectrophotometry (DU730, Beckman Coulter, Inc., Brea, CA, USA). ZnO NPs (MFD-2 ZnO, 0.5 and 1.0 mg⋅mL^−1^) and the premade NP aggregates (MFD-2 ZnO aggregates, 1.0 mg⋅mL^−1^) were prepared to suspend in 1 mL of hexane as samples. Conventional ZnO particles of nano-ZnO and hybrid ZnO in hexane at 1.0 mg⋅mL^−1^ were also used. Hexane was used as reference for background. UV-vis spectra were recorded at 190–800 nm.

### 2.11 Photocatalysis after sorption

For sorption, ZnO NPs at 0.01–1.0 mg⋅mL^−1^ were dispersed in 0.01 mg⋅mL^−1^ of aqueous methyl orange solution. After 60 min incubation in the dark, the sample absorbance levels were measured at 464 nm. After sorption equilibria, the samples were irradiated using a dual UV lamp of UV-A and UV-C in a CBD connected to an electronic controller at 40 W⋅m^−^^2^ [9]. The UV spectra of the lamp were acquired using a spectrometer (Jaz System, Ocean Optics, Inc., Orlando, FL, USA) with software (Spectra Suite, Ocean Optics, Inc.). UV dose (J⋅m^−2^) was calculated by multiplying the UV lamp intensity (9.31 W⋅m^−2^) with the exposure time (s). The UV-irradiated samples of methyl orange for 10 min were determined at 464 nm. Tests were also performed using ZnO NP aggregates. Briefly, the ZnO NP aggregates (1.0 mg) were suspended in 0.01 mg⋅mL^−1^ of aqueous methyl orange solution. After 30 min incubation in the dark, the ZnO NP aggregate samples were irradiated by the same UV lamp and collected at various time points of 10, 30, and 60 min. Next, the absorbance at 464 nm and the spectra of the samples were monitored using Synergy H1 hybrid multi-mode fluorescence reader (BioTek Instrumetns, Inc., Winooski, VT, USA). The reference wavelength was 600 nm.

### 2.12 Photoluminescence (PL)

ZnO NP suspensions at 0.75–6.0 mg⋅mL^−1^ were prepared in 1 mL of hexane. PL spectra of ZnO NPs were obtained at room temperature using Synergy H1 hybrid multi-mode fluorescence reader (BioTek Instrumetns, Inc.) with the excitation wavelength 358 nm and recorded using Gen 5 software (BioTek Instrumetns, Inc.). Emission wavelength was at a range of 400–700 nm.

### 2.13 Immobilization of microfluidically synthesized ZnO NPs on cellulose paper sheet depending on NP ethanol wetting and contents

Hexane suspensions of ZnO NPs (0.5 mg) were made to drop serially onto the cellulose filter paper. The hexane diluents of ZnO NPs were added onto the cellulose filter paper and heated at 50 °C for rapid evaporation. Two types of MFD-2 ZnOs (wMFD-2 ZnO in an ethanol-wetted NP form and dMFD-2 ZnO in a dried NP form) were used.

### 2.14 Brunauer-Emmett-Teller (BET) analysis for surface characterization

Specific surface area and meso-/micropore analyses of ZnO NPs were conducted based on the nitrogen adsorption-desorption isotherms with 1.0 h outgas at 300 °C. The experimental specific surface area, pore volume, and average pore diameter were calculated based on the BET theory.

## Ethics Statement

The work does not involve the subject of humans, animals, or data from social media platforms.

## CRediT authorship contribution statement

**Su-Eon Jin:** Conceptualization, Methodology, Data curation, Visualization, Investigation, Writing – original draft, Writing – review & editing. **Sung-Joo Hwang:** Conceptualization, Writing – review & editing. **Hyo-Eon Jin:** Conceptualization, Methodology, Writing – review & editing.

## Declaration of Competing Interest

The authors declare that they have no known competing financial interests or personal relationships which have or could be perceived to have influenced the work reported in this article.

## Data Availability

Dataset for hierarchical tetramodal-porous architecture of zinc oxide nanoparticles microfluidically synthesized via dual-step nanofabrication (Original data) (Mendeley Data).

## Appendix Supplementary Materials

Image, application 1

Image, application 2

Image, application 3

Image, application 4

Image, application 5

Image, application 6

Image, application 7

Image, application 8

Image, application 9

Image, application 10

Image, application 11

Image, application 12

Image, application 13

Image, application 14

Image, application 15

Image, application 16

Image, application 17

Image, application 18

Image, application 19
